# Comparison of Fluoroplastic Causse Loop Piston and Titanium Soft-Clip in Stapedotomy

**Published:** 2017-01

**Authors:** Mohammad Faramarzi, Nafiseh Gilanifar, Sareh Roosta

**Affiliations:** 1 *Otolaryngology Research Center, Shiraz University of Medical Sciences, Shiraz, Iran.*

**Keywords:** Fluoroplastic (Teflon) Causse loop piston, Otosclerosis, Stapedotomy, Titanium soft-clip

## Abstract

**Introduction:**

Different types of prosthesis are available for stapes replacement. Because there has been no published report on the efficacy of the titanium soft-clip vs the fluoroplastic Causse loop Teflon piston, we compared short-term hearing results of both types of prosthesis in patients who underwent stapedotomy due to otosclerosis.

**Materials and Methods:**

A total of 57 ears were included in the soft-clip group and 63 ears were included in the Teflon-piston group. Pre-operative and post-operative air conduction, bone conduction, air-bone gaps, speech discrimination score, and speech reception thresholds were analyzed.

**Results:**

Post-operative speech reception threshold gains did not differ significantly between the two groups (P=0.919). However, better post-operative air-bone gap improvement at low frequencies was observed in the Teflon-piston group over the short-term follow-up (at frequencies of 0.25 and 0.50 kHz; P=0.007 and P=0.001, respectively).

**Conclusion:**

Similar post-operative hearing results were observed in the two groups in the short-term.

## Introduction

The first stapes surgery was performed by Kessel in 1842 (1), although the first Teflon prosthesis was not used until 1956 when Shea pioneered the technique (2). Since the nineteenth century around 105 different types of prosthesis have been developed (3). The characteristics of acceptable stapes prostheses include the requirement that they produce a reasonable hearing result, are durable, do not cause an inflammatory reaction, and are secure to the long process of incus (4). Numerous materials and designs of stapes-replacement prostheses are currently available. Piston-style prostheses incorporating Teflon pistons with stainless steel or platinum wire loops are the two most common types (4,5). However, the new titanium prosthesis has a lower weight and is much softer, allowing for easier crimping around the incus (4). At our center, we have been using titanium soft-clip devices for the past few years. This device does not require crimping (one of the most difficult steps in stapes surgery) (6), but simply clicks on to the long process of the incus. There are numerous prostheses on the market, which suggest a lack of consensus with regards to the ideal prosthesis. Since there has been no documented report on the efficacy of a titanium soft-clip vs. a Teflon piston, we decided to compare short-term hearing results in patients who underwent stapedotomy with these prostheses.

## Materials and Methods

This single-blind, randomized clinical study was carried out on 148 ears among 110 patients. All patients underwent stapedotomy between October 2013 and March 2015 at Dasthgheib Hospital, affiliated with Shiraz University of Medical Sciences. All procedures were performed by the senior author. The research protocol was approved by Shiraz University Ethics Committee (RCT code: IRCT20140121 15496N5). Inclusion criteria were patients who suffered from otosclerosis and underwent primary stapedotomy in whom we used a fluoroplastic Causse loop piston (Medtronic Xomed Jacksonville, FL), or a titanium soft-clip (Kurz, Germany). Exclusion criteria were revision stapedotomy, stapedectomy due to other causes such as congenital stapes fixation, tympanosclerotic plaque, chronic otitis media, or trauma. In addition, patients who had inadequate follow-up (less than 6 months) were excluded from our study.

Patients were divided into the two groups by block randomization according to type of prosthesis. All operations were performed using a transcanal approach under local anesthesia. The footplate was drilled to a size of 0.7 or 0.6 mm by a powered microdrill for the Teflon-piston and soft-clip prostheses, respectively. The size of the Teflon-piston prosthesis was 4–6 mm in length and 0.6 mm in diameter and the soft-clip prosthesis was 4.25–4.75 mm in length and 0.5 mm in diameter. The site of the vestibulectomy was sealed with lobular fat.

In order to evaluate hearing results, pure tone audiometry at frequencies of 0.25, 0.5, 1, 2, and 4 kHz was analyzed. The audiologist who reviewed the hearing results was blind to the type of prosthesis used. As we usually do not evaluate the 3-kHz frequency in our center, we used the mean of 2- and 4-kHz frequencies instead of the 3-kHz frequency. In addition, pre-operative and post-operative air conduction (AC), bone conduction (BC), air-bone gaps (ABG), speech discrimination score (SDS), and speech reception thresholds (SRT) were analyzed. A pre-operative audiogram was performed 1 week prior to the surgery, and post-operative audiograms were analyzed at least 6 months after the surgery. SPSS software version 15 was used for statistical analysis. In order to compare categorical data we used the Chi-Square test. In addition, a paired t-test or Wilcoxon test was used to compare variables within the groups and an independent sample T-test or Mann-Whitney test was used to compare between-group variables. A p-value less than 0.05 was considered significant. 

## Results

**Fig 1:**
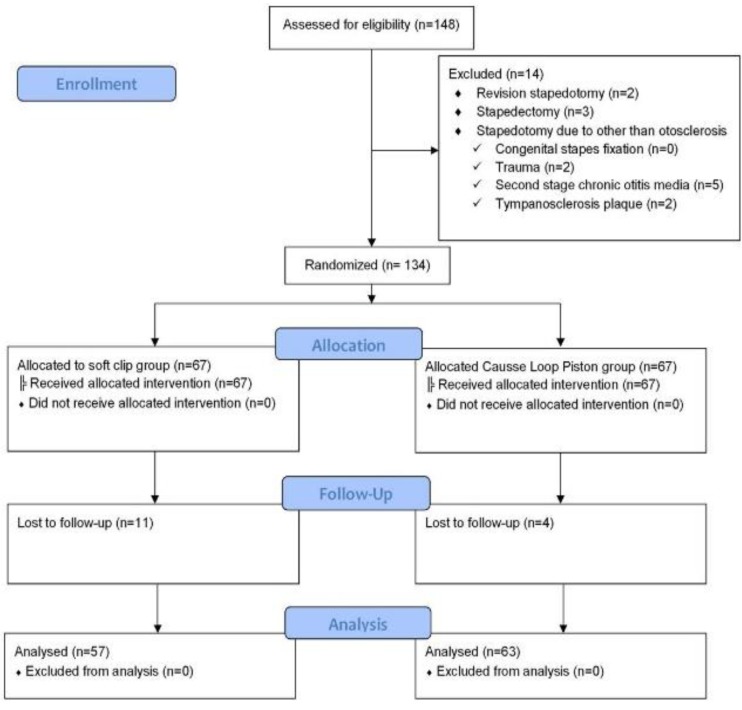
Consort trial flow diagram

Initially, 148 ears which had undergone stapedotomy were evaluated. As Figure 1 shows, 14 ears were excluded because of revision stapedotomy (two ears), stapedectomy (three ears), trauma (two ears), second stage chronic otitis media surgery (five ears), and tympanosclerosis plaque (two ears). The unequal number of ears within the groups was a result of patient withdrawal from the study. 

Eleven ears in the soft-clip and four ears in the Causse loop group were excluded. The Causse loop piston prosthesis was used in 63 stapedotomies. In this group, there were 32 (50.8%) men and 31 (49.2%) women, with a mean age of 34.1±11.2 years. The soft-clip prosthesis was used in 57 cases. This group consisted of 20 (35.1%) males and 37 (64.9%) females, with a mean age of 31.2±8.4 years. The two groups were homogenous with regards to gender (P=0.08) and age (P=0.114).

As Table 1 shows, the mean AC at measured frequencies of 0.5–3 kHz had improved postoperatively in each group (P<0.000). In addition, the mean AC gain at measured frequencies of 0.5–3 kHz was similar in both groups (P=0.653) (Table.1).

**Table 1:** Pre-operative and post-operative mean BC, AC and ABG (dB) in frequencies of 0.5–3 kHz.

		Pre-operative	Post-operative	Gain
Causse loop piston	Mean BC (dB)	15.0±8.5^a^	14.1±10.2	0.9±9.8
	Mean AC (dB)	49.0±10.6	31.9±14.6	17.1±16.1
	Mean ABG (dB)	34.1±9.2	17.8±7.6	16.3±11.0
Soft-clip	Mean BC (dB)	15.4±5.5	12.1±7.5	3.3±5.7
	Mean AC (dB)	46.1±7.7	30.3±11.1	15.9±12.7
	Mean ABG (dB)	30.8±7.9	18.1±6.4	12.6±9.8

a Values are Mean±SD, AC: Air Conduction, BC: Bone Conduction, ABG: Air-Bone Gap

We also compared mean BC gain at frequencies of 0.5–3 kHz between the two groups. Mean BC gain did not differ statistically between the groups (P=0.108) (Table.1). However, at frequencies of 0.25 and 0.50 kHz, mean BC gain showed a significant improvement in the soft-clip group compared with the Teflon-piston group (P=0.000, P=0.006,respectively). However, this difference was not clinically significant (Table.2).

**Table 2:** Post-operative AC, BC gain (dB) and ABG improvement (dB) in each frequency

		**250 Hz**	**500 Hz**	**1,000 Hz**	**2,000 Hz**	**3,000 Hz**	**4,000 Hz**
Causse loop piston	AC gain (dB)	19.2^a^	21.8	19.6	15.5	11.6	9.6
Soft-clip		15.9	17.6	18.3	14.3	13.3	14.3
P-value		0.274	0.155	0.669	0.663	0.543	0.144
Causse loop piston	BC gain (dB)	-0.1	-0.7	0.4	4.0	-0.3	-2.5
Soft-clip		4.1	3.0	3.0	4.1	2.8	-0.2
P-value		0.000	0.006	0.111	0.960	0.109	0.240
Causse loop piston	ABG improvement (dB)	19.3	22.5	19.2	11.5	11.9	12.1
Soft-clip		11.7	14.6	15.2	10.2	10.4	14.6
P-value		0.007	0.001	0.104	0.528	0.457	0.356

a Values are Means, AC: Air Conduction, BC: Bone Conduction, ABG: Air-Bone Gap

Significant improvement in post-operative ABG at frequencies of 0.5–3 kHz was seen in both groups (P<0.000). The improvement was greater in the Teflon-piston group in comparison with the soft-clip group (16.3 dB vs. 12.6 dB), but this difference was not statistically or clinically significant (P=0.060). ABG improvement was both clinically and statistically greater in the Teflon-piston group at frequencies of 0.25 and 0.50 kHz (P=0.007 and P=0.001, respectively) (Table.2).

Mean post-operative ABG at 0.5–3 kHz was categorized into four groups: ≤10, 11–20, 21–30,>30 dB. As shown in Table3, approximately 51 (80.9%) ears in the Causse loop piston group and 49 (86%) ears in the soft-clip group achieved a post-operative ABG of more than 20 dB. This difference was not statically significant (P=0.462).

Pre-operative and post-operative SDS and SRT are shown in Table.4. Post-operative SRT significantly improved using each type of prosthesis (P=0.000), although this improvement was not statically different between two groups (P=0.919) (Table.4). 

Furthermore, the improve- ment in post-operative SDS was not statistically 

significant between the two groups (P=0.747).

**Table 3:** Mean post-operative ABG distribution in frequencies of 0.5–3 kHz.

**ABG Closure**	**Causse loop piston**	**Soft-clip**
≤ 10	23(36.5)^a^	22(38.6)
11–20	28(44.4)	27(47.4)
21–30	8(12.7)	5(8.8)
> 30	4(6.3)	3(5.3)

a Values are N(%), ABG: Air-Bone Gap

**Table 4:** Pre-operative and post-operative SDS and SRT

		**Pre-operative**	**Post-operative**	**Improvement**
Causse loop piston	SDS (%)	92.5± 8.0^a^	94.0± 10.6	1.5 ± 12.8
	SRT (dB)	50.6± 11.0	31.8± 14.6	18.8 ±17.4
Soft-clip	SDS (%)	95.1± 4.0	96.0± 5.4	0.9 ±6.0
	SRT (dB)	48.0± 9.6	29.6± 10.3	18.5 ±14.4

a Values are Mean±SD, SDS: Speech Discrimination Scores, SRT: Speech Reception Thresholds

## Discussion

In this randomized clinical trial, we compared hearing results between the Teflon piston and the soft-clip. To the best of our knowledge, no other study has specifically compared the Teflon-piston with a titanium soft-clip. We found minimal difference between the two types of prosthesis, except for better post-operative ABG at a 0.25- and 0.50-kHz frequency in the Teflon-piston group. With respect to post-operative ABG within 10 dB, our results are within the range reported in the literature, which showed a 23–96% success rate (Table.5).

Both prostheses used in this study are non-crimping. Crimping is one of the most difficult stages in stapes surgery, with unpredictable results (11,14,16). It is assumed that tight crimping can lead to avascular necrosis. On the other hand, loose crimping can result in erosion of the long process of the incus (6,17). Therefore, if a prosthesis does not require crimping, this could potentially be an advantage over other types.

Even though the soft-clip prosthesis does not need manual crimping, the narrow opening at the anterior end of its loop means that it does need to be clicked onto the long process of the incus with a gentle push. Hence, if the force applied by the surgeon is excessive, it may dislocate the incus, leading to serious consequences in terms of hearing results. In contrast, due to the nature of Teflon, its loop spontaneously returns back to its original closed shape.

Thanks to modern technology, with the introduction of new biocompatible prostheses in the field of otology, otologists have the opportunity to trial various types of device; although there is conflicting evidence in the literature with regards to the superiority of one prosthesis over another. As shown in Table 5, there are numerous studies that have specifically compared different prostheses (5,7,15). For example, some authors have reported that a Nitinol prosthesis is a suitable prosthesis (4,5,15), while others believe that this prosthesis may increase the rate of revision surgeries (7). In another study, Brar et al. concluded that there were no differences between the Teflon-piston and a Nitinol piston (18). As shown in Table 5, Mangham et al. reported better results at low frequencies for non-crimping prostheses (7). However, others believe that there is no difference between crimping and non-crimping prostheses (15,19). Regarding the type of prosthesis, some studies have shown similar hearing results between other types of titanium prosthesis and the Teflon-piston (4,9). In contrast, other researchers reported better hearing results with a Teflon-piston prosthesis (10). Table 5 shows that, in general, most studies found no significant differences between the various prostheses.

The clinical finding of this study is that there is no difference between the soft-clip and fluoroplastic Causse loop piston with regards to post-operative ABG. One of the strengths of 

the current study is that all patients underwent the procedure by the senior author; hence, differences in the level of expertise may be excluded as a confounding factor. The second strength of this study was the prospective and randomized methodology employed. The only major drawback of this study was its short follow-up period.

**Table 5:** Literature review of post-operative hearing outcomes in stapes surgery regarding the type of prosthesis

**Author (yr )**	**Type of prosthesis** **(number)**	**Duration of follow-up**	**ABG ≤10dB** **(%)**	**ABG ≤20dB** **(%)**	**Conclusion**
Current study	Causse loop piston (63) Soft-clip (57)	At least 6 months	36.5 38.6	80.9 86.0	Similar good results with both prostheses. Causse loop piston was moderately better at low frequencies
Mangham (2010)(7)	Platinum piston (144) Nitinol–Teflon piston (44)	NR^a^	96 92	100 100	No significant difference in ABG closure (better mean ABG in lower frequencies in Nitinol–Teflon group)
Fayad et al (2009)(8)	SMart piston (306) Richards' Platinum piston (110)	5.6 months	78.3 84.2	94.2 98.0	Similar audiometric outcomes
Van Rompaey et a 2009)(9)	Teflon (211) Teflon wire (168) Titanium (112) Clip piston (49) Smart (74)	12 months	Overall 63.6	Overall 92.6	5 group had no difference in ABG closure
Mangham (2008 )(10)	Teflon piston 0.5 mm (74) Teflon piston 0.6 mm (74) Titanium clip piston (33)	1 year	85 91 84	NR	Teflon piston achieved better result than Titanium clip piston
Schrotzlmair et al (2013)(11)	Self -crimping Nitinol (Thermo Dummy) (21) Titanium K-piston (28) Clip piston a`Wengen (13)	70.7 days 89.6 days 163.1 days	76.2 53.6 23.1	95.2 89.3 69.2	ABG closure was better in self –crimping Nitinol especially in comparison with clip piston (a`Wengen)
Ying et al (2010)(12)	SMart (Teflon-based piston Nitinol) (190) Manual–crimp platinum; De La Cruz (145)	NR	NR	NR	11% Revision rate was seen in SMart group and 4% in De La Cruz group
Huber et al (2008)(13)	Conventional (75) Nitinol smart (75)	At least 12 months	43 71	92 94	Similar in ABG closure within 20 dB. better ABG closure within 10 db for Nitinol SMart
Tange and Grolman (2008 )(14)	Titanium K-piston (63) Clip piston a`Wengen (63)	NR	65 71	87.1 91	Similar audiometric outcomes
Brown and Gantz (2007)(5)	Platinum wire piston (39) Nitinol piston (40)	20 months 9 months	NR	NR	Similar audiometric outcomes
Massey et al (2005)(4)	Kurz titanium K- piston (35) Teflon platinum wire (183)	4 months	71 86	97.1 97.8	Similar audiometric outcomes
Zepeda-Lopez et al (2005)(15)	Schuknecht Teflon wire piston (70) Fluoroplastic Teflon (76)	NR	57.1 93.4	NR	Fluoroplastic Teflon had better ABG closure in all frequencies

a NR: not reported

## Conclusion

We found out that both the self-crimping fluoroplastic Causse loop piston and the soft-clip, which does not need manual crimping, yielded similar post-operative hearing results in the short-term. We conclude that, if an acceptable post-operative hearing result can be achieved with the appropriate expertise, it is not necessary to pursue the latest and most expensive types of prosthesis.
